# Supplementary material for: Patient-Reported Sexual Function, Bladder Function and Quality of Life for Patients with Low Rectal Cancers with or without a Permanent Ostomy
Source: Cancers (Basel). 2023 Dec 28;16(1):153. doi: 10.3390/cancers16010153 (PMC10778006; doi:10.3390/cancers16010153)

Supplemental Materials:

Supplemental Table S1. Characteristics of the study population

	Presence of Ostomy		
**Characteristic**	No (n = 25)	Yes (n = 22)	Overall (n = 47)
**Sex**			
Female	7 (28.0%)	9 (40.9%)	16 (34.0%)
Male	18 (72.0%)	13 (59.1%)	31 (66.0%)
**Race**			
Asian	2 (8.0%)	0 (0%)	2 (4.3%)
Black	2 (8.0%)	3 (13.6%)	5 (10.6%)
White	21 (84.0%)	19 (86.4%)	40 (85.1%)
**Ethnicity**			
Hispanic	3 (12.0%)	6 (27.3%)	9 (19.1%)
Non-Hispanic	22 (88.0%)	16 (72.7%)	38 (80.9%)
**BMI**			
Mean (SD)	29.5 (6.19)	30.0 (5.72)	29.7 (5.91)
Median [Q1, Q3]	29.6 [24, 33.6]	28.8 [25.6, 32.7]	29.3 [25.2, 33.2]
**Clinical T Stage**			
**1**	0	0	0
2	4 (16.0%)	3 (13.6%)	7 (14.9%)
3	19 (76.0%)	14 (63.6%)	33 (70.2%)
4	2 (8.0%)	5 (22.7%)	7 (14.9%)
**Clinical N Stage**			
0	9 (36.0%)	1 (4.5%)	10 (21.3%)
1	11 (44.0%)	15 (68.2%)	26 (55.3%)
2	5 (20.0%)	6 (27.3%)	11 (23.4%)
**Distance from the Anal Verge (cm)**			
Mean (SD)	3.45 (1.49)	3.40 (2.58)	3.43 (2.05)
Median [Q1, Q3]	3.5 [2.25, 5]	2.75 [2, 4.3]	3 [2, 5]
**EMVI**			
No	17 (68.0%)	12 (54.5%)	29 (61.7%)
Yes	8 (32.0%)	10 (45.5%)	18 (38.3%)
**RT Technique**			
3DCRT	16 (64.0%)	13 (59.1%)	29 (61.7%)
VMAT	9 (36.0%)	9 (40.9%)	18 (38.3%)
**RT Course**			
LCRT	18 (72.0%)	19 (86.4%)	37 (78.7%)
SCRT	7 (28.0%)	3 (13.6%)	10 (21.3%)
**Year of RT Completion**			
2017	6 (24.0%)	7 (31.8%)	13 (27.7%)
2018	6 (24.0%)	4 (18.2%)	10 (21.3%)
2019	5 (20.5%)	5 (22.7%)	10 (21.3%)

2020 and later	8 (32.0%)	6 (27.3%)	14 (29.8%)
**Surgery**			
No	13 (52.0%)	0 (0%)	13 (27.7%)
Yes	12 (48.0%)	22 (100%)	34 (72.3%)
**Sexually Active In last 30 days**			
No	7 (28.0%)	8 (36.4%)	15 (31.9%)
Yes	16 (64.0%)	13 (59.1%)	29 (61.7%)
Unknown/No response	2 (8.0%)	1 (4.5%)	3 (6.4%)

Supplemental Figure S1. Sexual (IIEF, FSFI) and urinary (MLUTS, FLUTS) patient-reported outcomes, with results displayed separately according to presence of an ostomy.

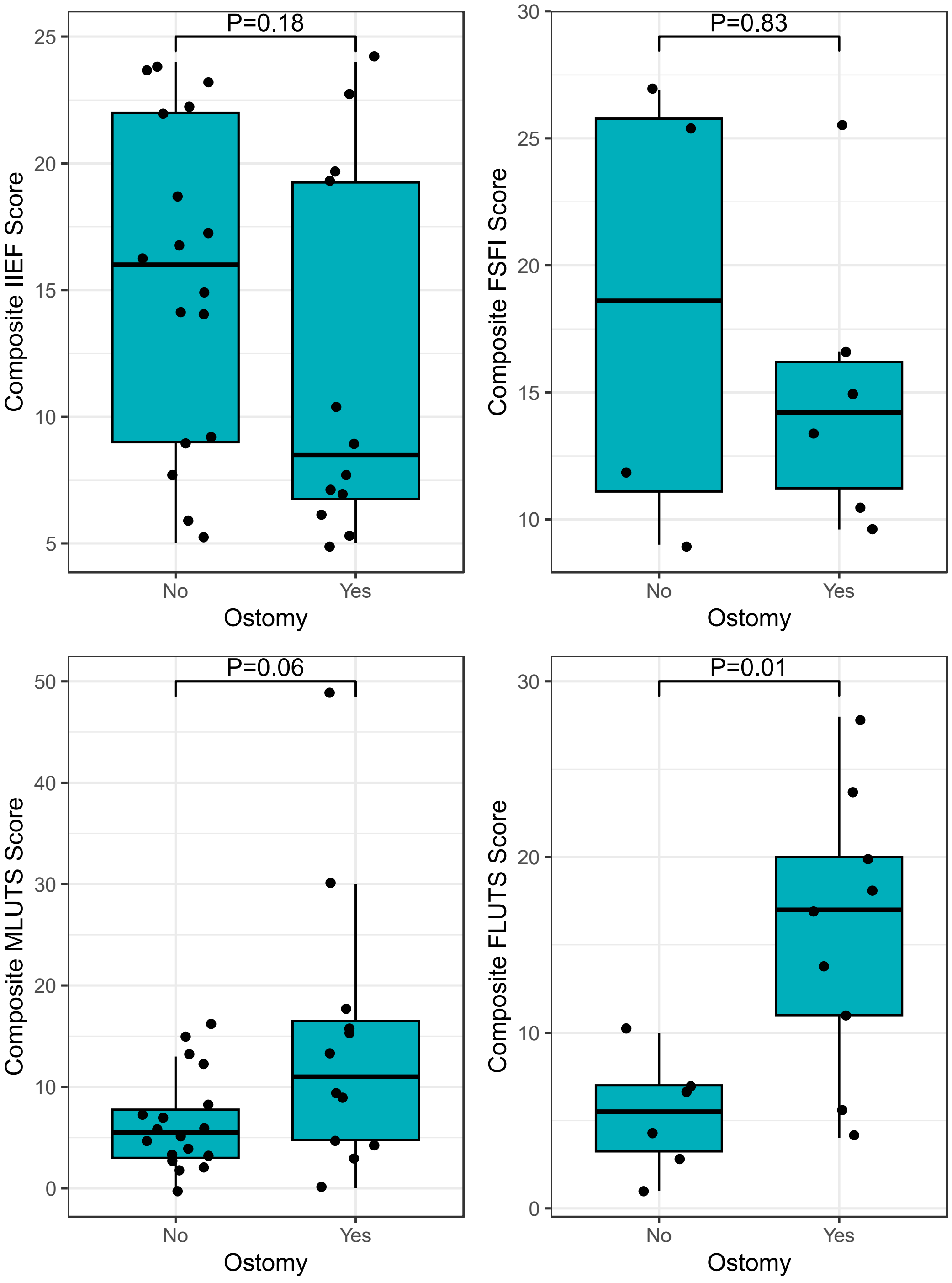

Supplement: Supplementary file 1 [file cancers-16-00153-s001.zip › cancers-2761520-supplementary.pdf]
